# Hematocrit and lactate trends help predict outcomes in trauma independent of CT and other clinical parameters

**DOI:** 10.3389/fradi.2023.1186277

**Published:** 2023-09-18

**Authors:** Pedro V. Staziaki, Muhammad M. Qureshi, Aaron Maybury, Neha R. Gangasani, Christina A. LeBedis, Gustavo A. Mercier, Stephan W. Anderson

**Affiliations:** ^1^ Department of Radiology, The University of Vermont Medical Center, Larner College of Medicine at the University of Vermont, Burlington, VT, United States; ^2^ Department of Radiology, Boston Medical Center, Boston University School of Medicine, Boston, MA, United States; ^3^ Department of Radiation Oncology, Boston Medical Center, Boston University School of Medicine, Boston, MA, United States; ^4^ Department of Radiology, Emory University, Atlanta, GA, United States

**Keywords:** trauma, computed tomography, hematocrit, intensive care unit, length of stay

## Abstract

**Background:**

Hematocrit and lactate have an established role in trauma as indicators of bleeding and cell death, respectively. The wide availability of CT imaging and clinical data poses the question of how these can be used in combination to predict outcomes.

**Purpose:**

To assess the utility of hematocrit or lactate trends in predicting intensive care unit (ICU) admission and hospital length of stay (LOS) in patients with torso trauma combined with clinical parameters and injury findings on CT.

**Materials and Methods:**

This was a single-center retrospective study of adults with torso trauma in one year. Trends were defined as a unit change per hour. CT findings and clinical parameters were explanatory variables. Outcomes were ICU admission and hospital LOS. Multivariate logistic and negative binomial regression models were used to calculate the odds ratio (OR) and incident rate ratio (IRR).

**Results:**

Among 840 patients, 561 (72% males, age 39 ± 18) were included, and 168 patients (30%) were admitted to the ICU. Decreasing hematocrit trend [OR 2.54 (1.41–4.58), *p* = 0.002] and increasing lactate trend [OR 3.85 (1.35–11.01), *p* = 0.012] were associated with increased odds of ICU admission. LOS median was 2 (IQR: 1–5) days. Decreasing hematocrit trend [IRR 1.37 (1.13–1.66), *p* = 0.002] and increasing lactate trend [2.02 (1.43–2.85), *p* < 0.001] were associated with longer hospital LOS.

**Conclusion:**

Hematocrit and lactate trends may be helpful in predicting ICU admission and LOS in torso trauma independent of organ injuries on CT, age, or admission clinical parameters.

## Introduction

As one of the leading causes of morbidity and mortality in the United States (US), trauma accounted for 671 billion dollars in medical expenses and lost productivity in 2013, encompassing 59% of deaths for people ages 1 through 44 (1). The 2016 annual report of the National Trauma Data Bank included over 800 thousand records of trauma in the US and described an overall mortality rate is 4.39% (2). Therefore, efficient care of trauma patients remains a top priority for hospitals in the US and worldwide.

There have been multiple models to help predict outcomes using data from national registries with many popular models, including anatomic scales, such as the Injury Severity Scale (ISS), and physiologic scales, such as the Revised Trauma Scale (RTS) and the Simplified Acute Physiology Scale-II (SAPS II), and combined anatomic and physiologic scales, such as the Trauma and Injury Severity Score (TRISS) (3). Also, there is increasing interest in using artificial intelligence to categorize patients using any available medical data, from vital signs to laboratory markers to medical images (4–6). Among commonly obtained laboratory exams in these patients, hematocrit and lactate have an established role in trauma. While hematocrit is an accurate indicator of bleeding (7), lactate is a known proxy for organ perfusion and cell death due to vascular injury or direct trauma (8). Finally, the wide availability of CT imaging and clinical data and the current pursuit of a “big data” approach for prediction pose the question of how clinical parameters can be used in combination with imaging to predict outcomes.

Our study aimed to evaluate the utility of hematocrit or lactate laboratory value trends in combination with organ injury by CT and other common clinical parameters to predict intensive care unit (ICU) admissions and hospital length of stay (LOS) of adult patients with chest, abdomen, or pelvis trauma.

## Materials and methods

### Patient identification

Our institutional review board approved this retrospective study, and consent was waived. All data were collected and stored in accordance with the Health Insurance Portability and Accountability Act using REDCap (Research Electronic Data Capture), the electronic data capture tool used in our institution (9).

We included all consecutive adult patients from 1 January through 31 December 2015 who received a trauma imaging protocol for blunt or penetrating trauma, queried from our hospital's trauma database. Exclusion criteria were age <16 years, any repeat exam, major head trauma resulting in intracranial hematoma, contusion or diffuse axonal injury, cervical fracture, facial fracture or spinal compression, or below-knee amputation. Additionally, for the analysis of LOS described below, we excluded patients who died during the same admission. This dataset and exclusions are the same as previously published [reference omitted for peer-review].

### Trends of hematocrit and lactate

We assessed trends of hematocrit and lactate for patients with at least two measurements in the first 30 h, including at admission. The first hematocrit and lactate measurements obtained are referred to as “baseline” throughout the manuscript and were also included in the analysis as a covariate in addition to the trend evaluation. First, hematocrit and lactate slopes (trends) were calculated for each patient with a linear regression analysis of serial measurements across time. Then, we converted continuous data into categorical data, with values > median used as a reference for hematocrit, since the opposite (decreasing hematocrit trend) is a surrogate of bleeding, and values ≤ median used as a reference for lactate, since the opposite (increasing lactate trend) is a surrogate of decompensating trauma. As a result, a trend of hematocrit ≤ median signifies a more rapid decrease in hematocrit level per hour, whereas a trend of lactate > median signifies a more rapid increase in lactate levels. In other words, the references were established to follow clinical rationale.

### Imaging and clinical covariates

Imaging data comprised the presence of injury to the lung parenchyma, ribs, liver, spleen, kidneys, colon, or bony pelvic ring detected by CT done in the trauma setting. These data were collected from each patient's Radiology report on the electronic medical record (EMR) and then subsequently reviewed by a radiologist blinded to clinical or outcome data. Clinical data were collected from the first 24 h of hospital stay, which preceded CT imaging and comprised admission systolic blood pressure (SBP), Glasgow Coma Scale (GCS), and hemoglobin. Demographic information of sex and age were also included. All clinical data represent the first data point, the initial value within 24 h.

### Outcomes

The outcomes of interest were admission to the ICU, defined as any medical or surgical ICU admission at any point during the hospitalization, and total hospital LOS in days.

### CT protocol

All CT examinations were performed with a 64–detector row CT scanner (Light-Speed VCT; GE Medical Systems, Milwaukee, WI). The following CT parameters were employed: reconstruction thickness, 0.625 and 1.25 mm; 120 kVp; noise index, 29 (automatic dose modulation); pitch, 1:0.984; and gantry rotation time, 0.5 s. In all cases, multiplanar reformations in coronal and sagittal planes were provided for interpretation (2.5 × 2.5 mm). Adaptive Statistical Iterative Reconstruction (40% ASIR; GE Medical Systems, Milwaukee, WI) was also implemented for trauma imaging.

Patients with abdominopelvic trauma at our institution initially receive a CT scan of the abdomen and pelvis following a standard 70-s delay (portal venous phase), acquired from the dome of the liver to the greater trochanters. Delayed phase images (5 to 7-min post-injection of contrast, pyelographic phase) through the abdomen and pelvis are also acquired at the discretion of a radiology resident or attending, who evaluate the initial portal venous phase images at the CT scanner at the time of acquisition. Delayed phase imaging is obtained to assess for active vascular contrast extravasation from vascular injury and contained vascular injuries such as pseudoaneurysms or arteriovenous fistulas or collecting system injury and allows for more definitive characterization of hyperattenuating foci seen on earlier acquisitions.

All patients presenting with penetrating trauma receive both oral and rectal contrast, in addition to intravenous contrast, per our departmental protocol. Patients presenting with blunt trauma do not receive any form of enteric contrast.

### Statistical analysis

Demographic characteristics, organ injury by CT imaging, and clinical parameters, available for all participants, are presented as frequency (percentage) for categorical data and mean ± standard deviation (SD) and median [interquartile ranges (IQR)] for continuous data.

Logistic regression models were employed to evaluate ICU admission, and crude and adjusted odds ratio (OR) with 95% confidence interval (95% CI) were computed. A negative binomial regression (NBM) model was fit to predict LOS by clinical and imaging factors. The NBM is the preferred model for skewed count data with overdispersion (mean and the variance are not equal), as is the case with our outcome data. Incident rate ratios (IRRs) were calculated with an IRR of >1 indicating an increase in LOS relative to its reference category. All analyses were two-sided, and *p*-values of less than 0.05 were considered statistically significant. All statistical computations were performed on SAS 9.1 system.

## Results

### Clinical and imaging characteristics

The initial query yielded 840 adult patients who underwent CT imaging for blunt or penetrating trauma from 1 January 2015 to 31 December 2015. From this cohort, 117 patients were excluded due to a combination of nondiagnostic exams or trauma outside of the chest, abdomen, or pelvis. Of these, an additional 162 patients were excluded due to a lack of hematocrit (2) or lactate (160) laboratory data in the first 24 h. After exclusions, 561 patients remained in the study, 72.7% male with a mean (STD) age of 39.1 (18.4) years. Five patients were excluded from the LOS analysis due to death during admission.

**Figure 1:**
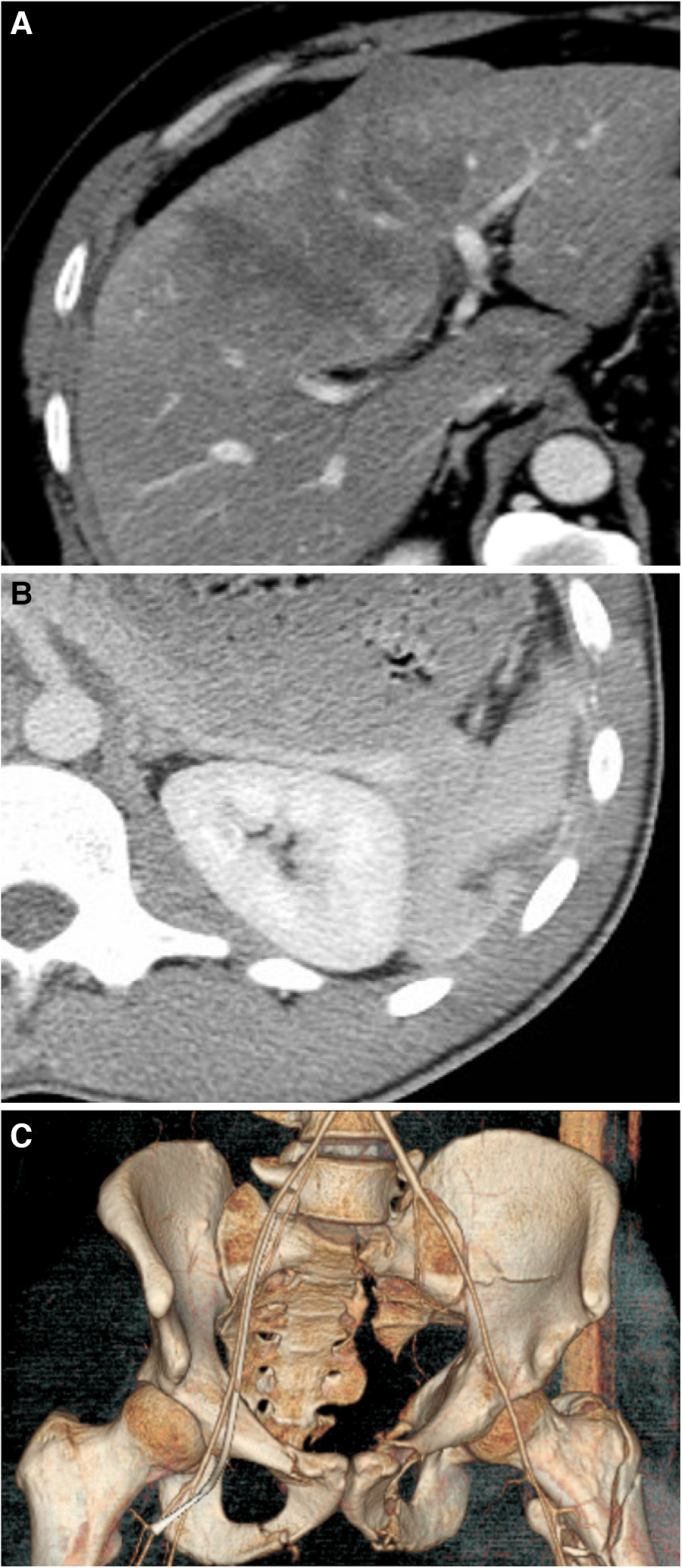
(**A**) A CT of the abdomen and pelvis showing a liver injury characterized by a subcapsular hematoma involving 10%–50% of surface area and an intraparenchymal hematoma measuring ≤10 cm in length (AAST grade II); (**B**) A CT of the abdomen and pelvis with contrast showing a splenic laceration of measuring less than 3 cm (AAST grade II); and (**C**) A 3D reconstruction of the pelvis of a 35-year-old man who fell from 5 stories showing multiple pelvic fractures, including the superior and inferior pubic ramus on the left, left acetabular fracture, and left moderately displaced sacral fracture, in addition to a left intertrochanteric fracture. There was also a suggestion of nondisplaced superior and inferior pubic rami fractures on the “right”.

All explanatory data, including age, sex, clinical parameters, trends of hematocrit and lactate, and clinical data, are described in Table 1. Rib and lung injuries were the most common finding, affecting 17.1% and 10.2% of patients, respectively. The average baseline hematocrit was 40.7%, and the average baseline lactate was 2.9 mmol/L. Figure 1 shows examples of trauma findings on CT.

**Table 1:** Descriptive characteristics.

	N	Mean	STD	Median	IQR	
Age (years)	561	39.1	18.4	35.0	24.0	51.0
Glasgow Coma Scale (GCS)	561	14.1	2.5	15.0	15.0	15.0
Hemoglobin (g/dl)	561	13.7	1.8	13.8	12.7	14.9
Hematocrit (%)	561	40.7	4.7	41.1	38.3	44.0
Lactate (mmol/L)	561	2.9	2.5	2.2	1.6	3.4
Systolic blood pressure (mmHg)	561	139.8	25.1	137.0	124.0	155.0
Length of stay (days)	556	4.8	7.8	2.0	1.0	5.0
Length of ICU stay (days)	166	4.9	6.1	3.0	2.0	5.0
Hematocrit trend	311	−0.29	0.30	−0.27	−0.44	−0.14
Lactate trend	145	−0.56	0.86	−0.23	−0.70	−0.05
	*n* (column percent)					
Gender						
Male	408 (72.7)					
Female	153 (27.3)					
ICU admission						
No	393 (70.1)					
Yes	168 (30.0)					
Lifesaving procedure						
No	530 (94.5)					
Yes	31 (5.5)					
Death						
No	556 (99.1)					
Yes	5 (0.89)					
Imaging findings						
Lung	57 (10.2)					
Rib	96 (17.1)					
Liver	27 (4.8)					
Spleen	23 (4.1)					
Kidney	21 (3.7)					
Colon	10 (1.8)					
Pelvis	51 (9.1)					

N, number of patients; STD, standard deviation; IQR, interquartile range; n, number of patients; ICU, intensive care unit.

Length of stay is missing for five patients who died during admission. Of 168 admitted to the ICU, the length of stay was missing for two patients.

### Trends of hematocrit and lactate

A total of 311 patients had two or more hematocrit laboratory measurements in the first 30 h; 166 patients had two values, 66 had three values, 33 had four values, 26 had five values, 17 had six, and three had seven values. The median trend of hematocrit was −0.27 percent per hour, i.e., a decrease of 1.35 percent units in five hours post-baseline. A total of 145 patients had two or more lactate laboratory measurements in the first 30 h, represented by 90 patients with two values, 23 with three values, seven with four values, five with five values, four with six values, seven with eight values, three with nine values, four with ten values two with 12 values. The median lactate trend was −0.23 units per hour, i.e., a decrease of 1.15 mmol/L in five hours post-baseline.

### ICU admission

Of the 561 patients, 168 (30%) patients were admitted to the ICU. In the multivariate model, including all patients (Model 1, Table 2), the clinical parameters that predicted admission to the ICU included age, GCS, and baseline lactate, whereas injuries to the lung [OR 3.29 (1.53–7.08), *p* = 0.002], rib [OR 4.11 (2.29–7.38), *p* < 0.0001], liver [OR 15.89 (4.81–52.5), *p* < 0.0001], spleen [OR 15.94 (4.04–62.82), *p* < 0.0001] and pelvis [OR 3.4 (1.54–7.49), *p* = 0.002] were independently associated with ICU admission.

**Table 2:** Multivariate logistic regression models for odds of ICU admissions.

	All patients (*N* = 561)		Hematocrit (*N* = 311)		Lactate (*N* = 145)	
	OR (95% CI), *p*					
Clinical parameters						
Age (years)	1.03 (1.02–1.05)	<0.0001	1.03 (1.01–1.05)	0.0004	1.02 (1.0–1.05)	0.071
Glasgow Coma Scale (GCS)	0.67 (0.58–0.76)	<0.0001	0.68 (0.58–0.80)	<0.0001	0.73 (0.60–0.88)	0.001
Hemoglobin (g/dl)	1.42 (0.87–2.33)	0.163	1.48 (0.84–2.61)	0.171	1.25 (0.55–2.87)	0.593
Hematocrit (%)	0.88 (0.73–1.06)	0.169	0.87 (0.71–1.08)	0.215	0.94 (0.68–1.30)	0.722
Lactate (mmol/L)	1.13 (1.04–1.23)	0.006	1.0 (0.90–1.11)	0.957	1.06 (0.91–1.24)	0.448
Systolic blood pressure (mmHg)	1.0 (0.99–1.01)	0.852	1.0 (0.99–1.01)	0.640	0.99 (0.97–1.01)	0.255
Hematocrit trend						
≤median −0.27 (range −1.72 to −0.27)			2.54 (1.41–4.58)	0.002		
>median −0.27 (range −0.26 to 1.05)			Reference			
Lactate trend						
≤median −0.23 (range −4.60 to −0.24)					Reference	
>median −0.23 (range −0.22 to 0.25)					3.85 (1.35–11.01)	0.012
Imaging findings (ref = no finding)						
Lung	3.29 (1.53–7.08)	0.002	3.50 (1.51–8.12)	0.004	2.45 (0.72–8.28)	0.150
Rib	4.11 (2.29–7.38)	<0.0001	2.90 (1.51–5.56)	0.001	2.02 (0.60–6.87)	0.258
Liver	15.89 (4.81–52.50)	<0.0001	10.12 (3.0–34.12)	0.0002	4.53 (0.70–29.39)	0.114
Spleen	15.94 (4.04–62.82)	<0.0001	7.50 (1.97–28.57)	0.003	2.18 (0.40–11.76)	0.365
Kidney	1.06 (0.33–3.43)	0.929	0.91 (0.28–2.98)	0.870	0.72 (0.09–6.18)	0.767
Colon	0.23 (0.04–1.40)	0.111	0.18 (0.03–1.05)	0.056	0.13 (0.02–1.06)	0.057
Pelvis	3.40 (1.54–7.49)	0.002	2.13 (0.90–5.04)	0.085	2.10 (0.56–7.91)	0.274

All patients: model including all patients; Hematocrit: model accounting for the trend of hematocrit, a smaller cohort of 311 patients with at least two hematocrit values; Lactate: model accounting for the trend of lactate, a smaller cohort of 145 patients with at least two lactate values; OR, ratio; CI, confidence interval; ref, referent.

When accounting for the trend of hematocrit in the multivariate analysis, a smaller cohort of 311 patients was included in the multivariate analysis, representing patients with at least two hematocrit values (Model 2, Table 2). A trend in hematocrit ≤ median was associated with increased odds of the ICU admission [OR 2.54 (1.41–4.58), *p* = 0.002], accounting for other clinical parameters and imaging findings. Age, GCS, injuries to lung, rib, liver, or spleen predicted ICU admission. In the analysis of lactate trend on 145 patients with at least two lactate values (Model 3, Table 2), a trend > median in the first 30 h was associated with increased odds of the ICU admission [OR 3.85 (1.35–11.01), *p* = 0.012], accounting for other clinical parameters and imaging findings. Only GCS remained statistically significant.

### Total hospital LOS

The overall median LOS in the hospital was two days (IQR: 1–5). For LOS analysis, five patients were excluded due to death during admission. In the multivariate NBM regression including all patients (Model 1, Table 3), lung [IRR 2.12 (1.62–2.76), *p* < 0.0001], rib [IRR 1.29 (1.04–1.60), *p* = 0.019], spleen [IRR 1.54 (1.05–2.25), *p* = 0.026], colon [IRR 1.93 (1.14–3.26), *p* = 0.014] and pelvis [IRR 2.60 (2.03–3.33), *p* < 0.0001] injuries in addition to age, GCS, hemoglobin, baseline hematocrit, and baseline lactate were associated with LOS in the hospital.

**Table 3:** Multivariate negative binomial regression model for length of stay.

	All patients (*N* = 556)		Hematocrit (*N* = 308)		Lactate (*N* = 142)	
	IRR (95% CI), *p*					
Clinical parameters						
Age (years)	1.01 (1.01–1.02)	<0.0001	1.01 (1.0–1.01)	0.038	1.0 (1.0–1.01)	0.285
Glasgow Coma Scale (GCS)	0.91 (0.88–0.94)	<0.0001	0.92 (0.90–0.95)	<0.0001	0.95 (0.91–0.98)	0.005
Hemoglobin (g/dl)	1.29 (1.10–1.51)	0.001	1.22 (1.03–1.45)	0.027	1.13 (0.87–1.46)	0.354
Hematocrit (%)	0.88 (0.83–0.94)	<0.0001	0.90 (0.84–0.96)	0.001	0.92 (0.83–1.01)	0.092
Lactate (mmol/L)	1.09 (1.05–1.12)	<0.0001	1.04 (1.0–1.08)	0.031	1.08 (1.03–1.14)	0.003
Systolic blood pressure (mmHg)	1.0 (1.0–1.0)	0.460	1.0 (1.0–1.0)	0.593	1.0 (1.0–1.0)	0.106
Hematocrit trend						
≤median −0.27 (range −1.72 to −0.27)			1.37 (1.13–1.66)	0.002		
>median −0.27 (range −0.26 to 1.05)			Reference			
Lactate trend						
≤median −0.23 (range −4.60 to −0.24)					Reference	
>median −0.23 (range −0.22 to 0.25)					2.02 (1.43–2.85)	<0.0001
Imaging findings (ref = no finding)						
Lung	2.12 (1.62–2.76)	<0.0001	1.67 (1.27–2.20)	0.0002	1.62 (1.11–2.36)	0.012
Rib	1.29 (1.04–1.60)	0.019	1.16 (0.93–1.44)	0.192	1.10 (0.77–1.58)	0.588
Liver	1.41 (0.98–2.02)	0.064	1.22 (0.87–1.71)	0.252	1.19 (0.74–1.90)	0.473
Spleen	1.54 (1.05–2.25)	0.026	1.22 (0.85–1.74)	0.273	1.38 (0.88–2.16)	0.156
Kidney	1.43 (0.96–2.13)	0.075	1.25 (0.86–1.82)	0.251	1.46 (0.83–2.55)	0.187
Colon	1.93 (1.14–3.26)	0.014	1.67 (1.0–2.79)	0.048	1.40 (0.81–2.43)	0.224
Pelvis	2.60 (2.03–3.33)	<0.0001	2.04 (1.60–2.62)	<0.0001	1.94 (1.39–2.71)	<0.0001

All patients: model including all patients; Hematocrit: model accounting for the trend of hematocrit, a smaller cohort of 311 patients with at least two hematocrit values; Lactate: model accounting for the trend of lactate, a smaller cohort of 145 patients with at least two lactate values; SE, standard error; ref, referent.

When accounting for the trend of hematocrit in the multivariate analysis, 308 patients were included, representing patients with at least two hematocrit values (Model 2, Table 3). A trend in hematocrit ≤ median was associated with a longer LOS [IRR 1.37 (1.13–1.66), *p* = 0.002], accounting for other clinical parameters and imaging findings. Patients with a hematocrit trend ≤ median stay 37% longer than patients with a hematocrit trend > median. Beyond trend in hematocrit, Model 2 also retained age, GCS, hemoglobin, baseline hematocrit, baseline lactate, and CT detected injuries (lung, colon, and pelvis) as significant model parameters. For lactate trend analysis (Model 3, Table 3), a trend in lactate > median in the first 30 h was positively associated with LOS [IRR 2.02 (1.43–2.85), *p* < 0.0001], accounting for other clinical parameters and imaging findings. Only lung and pelvic injuries remained statistically significant, with GCS and baseline lactate in Model 3.

## Discussion

This study retrospectively assessed the ability of trends in hematocrit and lactate in combination with organ injury identified at CT and other clinical parameters to predict clinical outcomes in trauma. We found that a decreasing trend in hematocrit and increasing trends in lactate are associated with increased odds of ICU admission and increased incident rate ratio of LOS, independent of imaging findings or other clinical parameters.

Both hematocrit and lactate are valuable predictors in the trauma setting. The rapid hematocrit changes after initiation of bleeding were shown in an animal model (10), and it can be a clinically useful predictor of bleeding (11) and significant injury (7, 12). Lactate is a known proxy for organ perfusion and cell death due to vascular injury or direct trauma. Hyperlactatemia in an emergency trauma patient suggests a high probability of severe injury (8). One study demonstrated that prehospital lactate level was higher in pediatric trauma patients who required critical care, including those who had normal prehospital vital signs and GCS. Other studies showed that prehospital lactate measurements improve the prediction of mortality, surgery, and multiple organ dysfunction syndrome (13).

Research has also been done to evaluate the usefulness of vital signs to enhance diagnosis and outcome prediction. One study assessed vital sign trends over 21 min and found these trends are unlikely to be diagnostically valuable because of substantial fluctuations across time, especially in higher-acuity patients (14). However, given the number of data points obtained in the hospital, we can potentially use this excess of data to predict outcomes in acute emergencies (15). The clinical parameters included in our models are similar to ones established in a large, validated model of mortality based upon data from the National Trauma Data Bank. In their 2014 study, Haider et al. found that a 6-covariate model including age, hypotension, pulse, GCS score, injury severity score, and need for ventilator use was ideal for predicting mortality amongst trauma patients (2). Given that the National Trauma Data Bank comprises nearly 700,000 unique data points a year, the shared identification of GCS and age as covariates lends credence to our findings. While our omission of injury severity score and need for ventilator use can be explained by their absence from our data collection process, our study may have been underpowered in comparison to derive significant predictive value from blood pressure and pulse.

There is no shortage of predictive analytic studies in the trauma literature. However, only a few include imaging. For example, a review by Tran et al. looking at models for predicting massive transfusion requirements examined 21 models, of which only 3 included CT findings (16). While this partially reflects the specific question Tran et al. were examining, it does demonstrate a need to integrate imaging findings into future model development. In addition, it is worth noting that the association of LOS or ICU admission with CT imaging findings disappears after adding lactate trend to the model. This could mean that, in the absence of the trend, which can take a few hours to collect, the imaging could be a surrogate for the association between ICU admission and LOS and the trend in lactate. However, once the trend is defined, the imaging carries less value. This demonstrates the value of adding CT imaging findings to clinical data models.

Adding new imaging features to clinical parameters could help improve their prognostic value. For instance, while CT IVC diameter (17), psoas muscle CT density (18), and dynamic CT washout of the renal medulla (19) have all been validated as independent predictors of mortality in various trauma and shock states, none have been combined with other clinical data. By combining these findings with other parameters, new clinical support tools with improved sensitivity and specificity can be developed. Regarding ICU admission, it is no surprise that increasing hematocrit trend is associated with decreased odds whereas increasing lactate trend is associated with increased odds of ICU admission. As expected, increasing hematocrit is a good sign in the trauma setting, which is the opposite of lactate, as increasing lactate signifies worsening illness.

Unfortunately, only a few published studies look at admission to ICU as an outcome of trauma. One study found that the ISS has a superior ability to predict both ICU admission and hospital LOS compared to the New ISS (NISS), which is calculated as the sum of squares of the three most severe injuries regardless of the body region injured (20). While we limited our approach to only logistic regression, greater accuracy could have been achieved by utilizing machine learning methods. Recently, Churpek et al. compared various regression and machine learning methods utilizing clinical parameters from the wards to predict ICU transfer, noting the superiority of machine learning models that displayed greater areas under the receiver operator characteristic curves than the regression models (21). Further research integrating imaging findings into these models may power them further, enabling even more reliable prediction of intensive care needs.

As for hospital LOS, we found a similar pattern: increasing hematocrit trend correlated with shorter LOS, whereas increasing lactate correlated with longer LOS. Again, this reflects the behavior of hematocrit and lactate in the trauma setting, with hematocrit decreasing in bleeding and lactate increasing in illness. A study found that sustaining at least five rib fractures in older patients with trauma is a significant predictor of intensive care unit admission and hospital stay (22). In 2015, Gholipour et al. utilized an artificial neural network trained on TRISS components, biochemical data, risk factors, and outcomes to predict ICU LOS and LOS with 93.3% accuracy (23). While inclusive of head and neck trauma, their model reflects the incredible power of new statistical approaches to augmenting traditional risk stratification scores for trauma patients. Future neural network development utilizing imaging findings in conjunction with biochemical and clinical data may increase diagnostic accuracy for targets as elusive as LOS and LOS ICU.

Limitations of this study include its retrospective design and small size. Most importantly, this was a sample of convenience with an inherent selection bias, as subjects with worse trauma or illness undergo more and more frequent laboratory testing than those with milder trauma, and these were the patients included in the trend analysis. Also, the measurements of hematocrit and lactate were not obtained at pre-specified intervals. Rather, we took any measurement obtained in the first 30 h, which would have been different for each patient. Different times and degrees of fluid resuscitation may have been administered to patients within the 30 h when hematocrit and lactate data were obtained, which could have clearance of lactate and variation of hematocrit. Additionally, we did not include the presence or absence of specific pre-existing conditions or comorbidities that would impact the parameters that are being evaluated, such as smoking, diabetes, blood disorders (including anemia), and dehydration stations, among others. Although it is conceivable that comorbidities such as these could affect outcomes (for example, by prolonging ICU stay with extubation complications in the setting of smoking), we decided to focus on trauma-related complications, which could have an impact on outcomes at a greater scale. Of note, our model is based upon the experience of a single level 1 trauma center. While it identified age and GCS as important covariates shared by the trial analyzing covariates in the National Trauma Data Bank, our number of entries is comparatively small (*n* = 561 vs. *n* = 630,307), and thus could potentially be underpowered to detect significance. Unfortunately, large databases linking trauma imaging findings to clinical data are not easily accessible. Finally, we deliberately excluded neurotrauma in our analysis, making our results less generalizable since patients undergoing polytrauma are at high risk for neurotrauma and worse outcomes based upon devastating CNS injury.

In summary, we found that trends of hematocrit and lactate combined with organ injury by CT imaging and other clinical parameters helped predict ICU admission and increased LOS in the hospital. Specifically, a decreasing trend in hematocrit is associated with increased odds of ICU admission and incident rate ratio of the length of stay, independent of organ injury. At the same time, an increasing trend in lactate is associated with increased odds of ICU admission and incident rate ratio of the length of stay, independent of organ injury. This data highlights the intriguing possibility of integrating trends of laboratory values with CT imaging findings into clinical decision support mechanisms to refine prediction in the trauma setting. Additionally, lactate and hematocrit trends, along with imaging, could be prioritized and expedited in remote locations with limited resources.

## Data Availability

The raw data supporting the conclusions of this article will be made available by the authors, without undue reservation.
